# Functional Methods for Studying Sperm–Zona Pellucida Interaction in Mammals

**DOI:** 10.3390/mps8040095

**Published:** 2025-08-13

**Authors:** Natalie Zelenkova, Veronika Kraus, Alexandra Maresova, Zuzana Pilsova, Aneta Pilsova, Barbora Klusackova, Eva Chmelikova, Katerina Komrskova, Pavla Postlerova

**Affiliations:** 1 Department of Veterinary Sciences, Faculty of Agrobiology, Food and Natural Resources, Czech University of Life Sciences Prague, 165 00 Prague, Czech Republic; zelenkovan@af.czu.cz (N.Z.); xmara142@studenti.czu.cz (A.M.); pilsovaz@af.czu.cz (Z.P.); klusackovab@af.czu.cz (B.K.); chmelikova@af.czu.cz (E.C.); 2 Laboratory of Reproductive Biology, Institute of Biotechnology of the Czech Academy Sciences, BIOCEV, 252 50 Vestec, Czech Republic; veronika.kraus@ibt.cas.cz (V.K.); katerina.komrskova@ibt.cas.cz (K.K.); 3 Department of Zoology, Faculty of Science, Charles University, 128 00 Prague, Czech Republic

**Keywords:** sperm–ZP binding, sperm receptor, binding assay, antibody block, competitive assay, hemizona binding assay, knockout model, antibody, recombinant protein

## Abstract

The initial molecular events mediating mammalian sperm binding to the zona pellucida (ZP) of the oocyte are highly complex and still not fully elucidated. Recent advances have identified multiple candidate sperm surface proteins, often functioning as part of high-molecular-weight complexes that mediate this critical fertilization event in a species-specific and coordinated manner. To address a significant gap in the literature, we provide an in-depth overview of the functional assays employed to investigate sperm–ZP interactions, emphasizing their underlying principles, potential applications, and key methodological strengths and limitations. The techniques discussed range from classical in vitro sperm–oocyte and hemizona binding assays, including antibody-blocking and competitive strategies, to cutting-edge in vivo genetic models, each contributing unique insights into the physiological relevance of the proposed ZP receptors. Robust experimental design, including the use of appropriate controls and validation strategies, is essential for accurately interpreting the role of candidate sperm receptors. This review provides a structured overview of current methodologies to support researchers in critically evaluating and applying functional assays in future studies.

## 1. Introduction

The initial binding of mammalian spermatozoa to the oocyte’s zona pellucida (ZP) is a complex and not yet fully understood process. Several candidate sperm surface proteins mediating this interaction have been identified, particularly in mouse and pig models [1,2,3]. Despite the species-specific nature of sperm–ZP binding, orthologous or functionally analogous proteins have been sought across mammals [2]. However, some ZP-binding proteins, such as spermadhesins in pigs, appear to be species-specific and multifunctional [1]. The earlier view that a single receptor mediates sperm–ZP binding has been replaced by the idea that multiple low-affinity interactions act in coordination [4]. This multivalent strategy may enhance the likelihood of successful sperm attachment and fertilization [3,5,6]. This model would also explain the existence of numerous sperm receptor proteins that have been described to be involved in the sperm interaction with ZP, such as galactosyltransferase [7], which is the best described ZP receptor in mouse, acrosin [8], zonadhesin [9], arylsulfatase A [10], or zona receptor kinase in human [11]. The current generally accepted concept then assumes that the primary attachment of the sperm to the ZP of the oocyte involves a complex of many surface proteins, not just one receptor, and the sperm thus participates in multiple simultaneous or sequential binding events with ZP ligands. In many species, a significant number of plasma membrane proteins showing affinity for ZP have been described to date on the sperm head, and the list of these receptors is continually refined as new potential proteins for binding are discovered and the importance of others is disproved, for example, by knockout experiments [6]. These receptors cluster together to form high-molecular-weight complexes that appear to contain a variety of proteins with different functions, including chaperones, enzymes, signaling proteins, and cell adhesion proteins, thereby mediating the sperm interaction with the ZP [12]. ZP-binding complexes have been found assembling into lipid rafts in the anterior plasma membrane of the sperm head, and during sperm capacitation, these complexes undergo remodeling and redistribution [12,13,14,15].

Despite increasing knowledge, no review has systematically evaluated the methods used to validate these receptors. Because sperm–ZP binding involves a coordinated, multimolecular process, its study remains technically challenging. Techniques range from basic microscopy to advanced biochemical, molecular, and genetic approaches. Nevertheless, the roles of many candidate receptors remain poorly defined, supported by varying degrees of experimental evidence. This review does not aim to summarize all identified ZP receptors (see comprehensive review by Tumova et al. [16], but rather focuses on functional assays used to investigate sperm–ZP interactions, emphasizing their principles, applications, and methodological strengths and limitations.

## 2. Functional Sperm–ZP Binding Assays

Functional binding assays aim to determine the physiological role of specific proteins in sperm–ZP interaction. These assays evaluate the sperm–ZP interaction in vitro and quantify either the number of spermatozoa that bind or the degree of inhibition of binding. The principle of binding studies relies on measuring interactions between two molecules, typically detected using secondary systems such as immunofluorescence. These studies include direct binding assays, which assess whether sperm can bind to the ZP at all, a useful approach combined with knockout experiments discussed later [17,18]. Other studies employ more complex approaches involving antibodies or various competitors [19,20,21,22].

In these types of sperm–ZP assays, both sperm and ZP (ZP-intact oocyte, isolated ZP, or hemizonae) pretreatment approaches can be performed using either specific antibodies or competing antigens, each offering distinct advantages. Antibody-based ZP pretreatment can block specific epitopes on the oocyte surface, directly testing their role in sperm binding, while competing antigen pretreatment assesses whether saturating ZP-binding sites with soluble ligands prevents sperm attachment. Conversely, sperm pretreatment with antibodies enables the identification of functionally important surface proteins, but it may also alter sperm function. In contrast, incubating sperm with soluble competing ligands, such as recombinant ZP proteins or peptides, can inhibit receptor availability without the potential steric hindrance. While antibodies offer high specificity and can be used for mechanistic targeting, competing ligands provide a more physiologically relevant mimic of native interactions. Together, these complementary strategies enable the cross-validation of binding partners from both the oocyte and sperm sides, thereby enhancing the robustness of conclusions about the molecular mechanisms underlying sperm–ZP recognition.

### 2.1. Antibody Blocking Binding Assay

**Figure 1:**
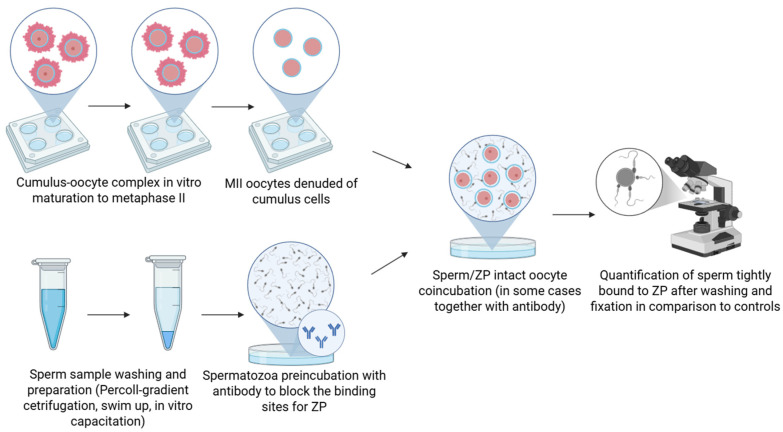
Schematic representation of the antibody block binding assay. Spermatozoa are preincubated with specific antibodies targeting the putative receptor, followed by exposure to isolated zona pellucida or zona-intact oocytes, or the incubation takes place during the sperm/oocyte coincubation. Inhibition of binding compared to controls suggests that the targeted protein is involved in the sperm–zona interaction. Created in BioRender. Aneta Pilsova (2025) https://BioRender.com/l3m8ngl (accessed on 30 June 2025).

The most commonly used method for studying sperm–ZP interaction is the antibody binding assay. The principle of this method involves the attachment of an antibody to a specific sperm surface protein during antibody pretreatment, either prior to or during sperm co-incubation with ZP-intact oocytes (Figure 1). If this protein mediates sperm binding to the ZP, the gamete interaction is either blocked or significantly reduced. Both monoclonal antibodies (mAbs), which consist of a single IgG molecule targeting a single epitope, and polyclonal antibodies (pAbs), which contain a mixture of IgG molecules targeting multiple epitopes on a given antigen, are routinely used. A key advantage of pAbs lies in their ability to recognize multiple epitopes on a single protein, enabling more effective blocking of functional domains required for sperm–oocyte binding. For instance, in studies of AWN spermadhesin, pAbs were significantly more effective, reducing sperm binding to the ZP by 87%, compared to 64% achieved by mAb. However, pAbs are generally less specific and may also react with proteins similar to the target protein. In contrast, mAbs exhibit high specificity toward a single epitope and typically do not recognize proteins other than their intended target. However, due to this specificity, their capacity to block binding may be weaker and less effective [20]. A limitation of the method is that antibody binding may result in steric hindrance, whereby the antibody physically obstructs sperm access to the ZP, which may appear as direct inhibition when evaluated.

Binding studies utilizing pAbs have been used to investigate the mouse protein SLIP1 [23], also known as P68, which was later found to be identical to arylsulphatase (AS-A) [10]. Pre-incubation of sperm with anti-SLIP1 antibodies resulted in a decrease in sperm binding to the oocyte. The level of inhibition was positively correlated with the antibody concentration [23]. Sperm pretreatment with pAbs prior to binding assay helped to identify sulfogalactosylglycerolipid (SGG) as a key sperm molecule involved in ZP binding. Sperm were incubated with anti-SGG antibodies and subsequently with oocytes, resulting in a reduction in the number of bound sperm [24]. The same binding assay was later employed to study the role of SLIP1 in human sperm–ZP binding, demonstrating that pretreatment of human spermatozoa with anti-SLIP1 IgG resulted in a significant reduction in the number of sperm bound to the ZP compared to controls treated with normal rabbit serum IgG. Importantly, this decrease in binding was not attributed to changes in sperm motility or an increase in premature acrosome reaction (AR), indicating a specific role of SLIP1 in ZP binding [25].

The involvement of sperm surface protein arylsulphatase (AS-A) in sperm–ZP binding was evaluated using mouse and pig in vitro binding assays. In mice, sperm preincubated with varying concentrations of anti-AS-A IgG or Fab fragments exhibited a dose-dependent reduction in ZP binding, with up to 70% inhibition at an antibody concentration of 100 µg/mL. Comparable effects were observed using affinity-purified antibodies, confirming specificity for AS-A and indicating that the inhibition resulted from direct masking rather than steric hindrance [10]. The same assay was also used to investigate the AS-A role in pigs by Carmona et al. [26]. Capacitated boar sperm were incubated with anti-AS-A IgG, Fab fragments, or control IgGs. Pig sperm pretreated with anti-AS-A antibodies showed a dose-dependent inhibition of sperm–ZP binding, confirming AS-A’s functional involvement. Anti-AS-A Fab fragments also inhibited binding, but to a lesser extent, suggesting specific masking of AS-A [26].

The importance of choosing the appropriate concentration of the antibody can be illustrated by the study of the 55 kDa boar sperm protein. Capacitated sperm were incubated with anti-55 kDa protein antibodies, washed, and incubated with oocytes, and sperm binding to the ZP was evaluated. Preincubation of sperm with anti-55 kDa antibodies reduced binding in a dose-dependent manner [27].

A complex approach was taken to evaluate the function of the pB1/DQH, which is a boar seminal plasma protein belonging to the Binder Sperm Proteins with fibronectin domain II, attached to the sperm surface during ejaculation and has an affinity to ZP glycoproteins [28]. First, it was investigated whether blocking ZP-binding sites with DQH antigen or mAb affects sperm binding. Oocytes were incubated in droplets with DQH/G12 antibody, DQH antigen, both antibodies and antigen, or medium only as a control. In another set of experiments, pretreatment of sperm with antibodies was performed to test whether coating sperm with antibodies (DQH/G12 and DQH/H6) inhibits their ability to bind the ZP. Control groups consisted of sperm preincubated in Sp2/0 ascitic fluid (myeloma cell line) and untreated sperm [29].

Another study utilized mAb ACR.2 (against acrosin) and Hs-8, which recognizes an intra-acrosomal protein, produced in the laboratory by immunizing mice with human ejaculated sperm. When added before capacitation of boar sperm, ACR.2 and Hs-8 had no effect on surface sperm proteins or sperm binding to the oocyte. However, when the antibodies were present during sperm–oocyte co-incubation, they significantly inhibited binding, suggesting a role in post-acrosomal binding events, possibly through interaction with exposed acrosomal proteins [30]. Margaryan et al. [21] also tested the effect of mAb Hs-8. Pretreated spermatozoa with Hs-8 (recognizing GAPDHS) and commercial anti-GAPDHS were co-incubated with oocytes. The presence of these antibodies (undiluted hybridoma supernatant, immunoglobulin concentration < 20 μg/mL) reduced the number of bound sperm to 18% and 21%, respectively, compared to the control group without antibodies. As a positive control using ACR.2 antibody, which recognizes porcine acrosin, the number of bound spermatozoa was reduced by 25% [21].

Studies in mice have assessed acrosomal or epididymal proteins using similar experimental designs involving capacitated sperm and pAbs. For example, anti-CRISP1 antibodies significantly inhibited sperm binding to ZP-intact eggs, while controls with normal rabbit IgG had no effect [31]. Likewise, anti-SED1 antibodies drastically reduced sperm–ZP adhesion compared to robust binding in control groups [32].

Galactosyltransferase (GalTase), a well-characterized sperm receptor for ZP3 in mice [7,33], was studied as a conserved gamete receptor across mammalian species. In bovine sperm, GalTase was localized to the anterior head region, and its functional role was demonstrated by a significant reduction in sperm–oocyte binding and fertilization following antibody blocking. The binding decreased by more than half compared to pre-immune serum [34].

Secondary ZP receptors were studied using antibody-blocking assays after inducing the AR. Binding studies were also performed with mAb 1A1 against human acrosomal antigen in mouse, pig, and human models, showing species-wide reduction in sperm–ZP binding [35]. In studies with bull sperm, anti-SPAM1 antibodies targeting either N- or C-terminal domains were used to evaluate binding to ZP. Blocking the C-terminal domain reduced binding by ~85%, compared to ~30% inhibition with N-terminal antibody [36]. In guinea pigs, sperm treated with anti-PH-20 (hyaluronidase, SPAM1) antibody post-AR showed minimal ZP binding, compared to untreated sperm [37]. SP-10 function was studied during bovine in vitro fertilization (IVF) using sperm–ZP binding assays and antibody-treated sperm. While untreated or control sperm showed firm ZP binding, sperm exposed to SP-10 mAbs bound only superficially, indicating impaired secondary binding [38].

The same assay can also be used to investigate receptor complexes. Redgrove et al. [39] demonstrated that blocking components of high-molecular-weight protein complexes, such as CCT6A or ZPBP2, reduced sperm–ZP binding in human gametes. Similarly, Cohen and Wassarman [40] demonstrated that mouse sperm vesicles enriched in sp56 protein effectively inhibited sperm–ZP binding. Pre-treatment with anti-sp56 antibody reduced binding by ~70–80%, and vesicles preincubated with anti-sp56 lost their inhibitory effect, confirming the functional role of sp56. Anti-human mAb C5F10, targeting the proacrosin/acrosin polysulfate-binding domain, inhibited human sperm–ZP binding without affecting fertilization of zona-free hamster eggs, confirming specificity [41]. Together, these cross-species studies reinforce the validity of using this method to identify and functionally characterize key sperm surface proteins involved in fertilization.

Many other studies used this method for assaying various potential sperm ZP receptors, for example, mouse hyaluronic acid binding protein (HABP) [42], acrosin-binding protein in pig (ACRBP) [43], P34H in human [44], hamster [45], and mice [46], α-L-fucosidase [47], or SLIP1/AS-A [48]. Key studies that have employed antibody block binding assays are summarized and described in more detail in Table 1.

Numerous studies emphasize the importance of a well-designed experimental setup in antibody-blocking assays. Variability in antibody type (polyclonal, monoclonal, purified vs. hybridoma supernatants), concentration, and epitope location can significantly affect the outcomes. For instance, hybridoma supernatants may contain undefined serum proteins or enzymes that influence sperm function independently of antibody specificity [21]. To verify the specificity of observed effects, most studies include controls such as preimmune serum, isotype-matched IgGs, and medium-only treatments. Furthermore, it is essential to assess sperm motility, viability, and acrosomal status following exposure to antibodies [25,41,49]. Quantification methods vary: some studies count all sperm attached to the oocyte, while others assess only those on one hemisphere [34,37] or in the focal plane of the ZP diameter [26,50], which may introduce inconsistencies and potential biases in interpretation and compromise cross-study comparisons. Antibody-blocking sperm–ZP binding assays have been widely used across mammalian species to functionally assess the involvement of specific sperm proteins in gamete recognition. While the core principle, preincubation of spermatozoa with antibodies followed by co-incubation with ZP-intact oocytes, remains consistent, studies differ in terms of antibody type, control design, quantification methods, and experimental parameters.

### 2.2. Competitive Binding Assay

**Figure 2:**
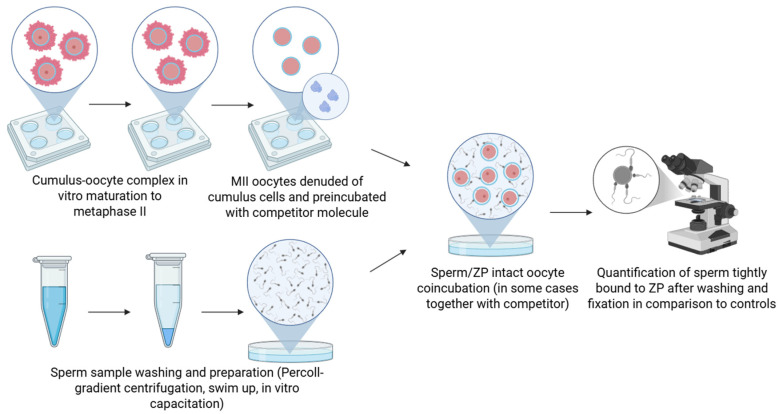
Schematic representation of the competitive binding assay. ZP-intact oocytes or isolated ZP are incubated with soluble or recombinant forms of the putative receptor or its ligand domain prior to exposure to spermatozoa, or the incubation takes place during the sperm/oocyte coincubation. A reduction in sperm binding compared to controls indicates competitive inhibition and supports the candidate’s role in sperm–zona recognition. Created in BioRender. Aneta Pilsova (2025) https://BioRender.com/l3m8ngl (accessed on 30 June 2025).

The competitive binding assay was designed to evaluate the involvement of specific molecules in sperm–ZP interaction by introducing soluble ligands, such as proteins, peptides, or carbohydrates, during sperm–oocyte co-incubation. These competitors are presumed to bind to ZP or sperm binding sites, thereby preventing native sperm surface molecules from interacting with the oocyte. A significant reduction in sperm binding indicates that the competitor mimics or blocks a functional binding domain. In competitive binding studies aimed at assaying the potential sperm ZP-receptor proteins, ZP-intact oocytes are typically preincubated with a competitor molecule that corresponds to the sperm receptor. Once the oocyte surface is saturated with the protein, any unbound protein is washed away, followed by the addition of the spermatozoa (Figure 2). A control group, comprising sperm and oocytes without any added competitor molecules, is also included. After incubation, the number of bound spermatozoa is evaluated. A reduction in sperm binding indicates that the tested protein inhibited binding, suggesting that the antigen possesses receptor activity. Conversely, if no inhibition occurs, the tested protein is likely not essential for binding [51].

In pigs, the spermadhesins AQN-1, AWN-1, and AWN-2 were studied using ZP-intact oocytes preincubated with isolated protein before sperm addition. Oocytes treated with AQN-1 showed a concentration-dependent reduction in sperm binding [19,51], and similar inhibitory effects were confirmed for AWN-1,2 in a dose-dependent manner [52]. Similarly, for another seminal plasma protein, co-incubation of capacitated boar spermatozoa with oocytes in the presence of pB1/DQH protein in the culture medium also strongly reduced sperm binding, and the percentage of zona-bound spermatozoa decreased to 14% [29]. Likewise, SLIP1 (AS-A) was shown to inhibit sperm–ZP interaction when added exogenously, either to sperm suspensions or directly to the oocytes or isolated ZP, confirming its dual capacity to block sperm and ZP binding sites. Denaturation of SLIP1 abolished its function, underlining the importance of native protein conformation [23].

In cattle, competitive binding studies evaluated SPAM1 (PH-20), showing that preincubation of oocytes with purified SPAM1 reduced sperm–ZP binding by up to 50% at 100 ng/mL and even more at 1 μg/mL. Deglycosylation of SPAM1 had no effect on its inhibitory activity, suggesting that glycosylation is not required for its binding to the ZP [36]. A similar strategy was used in water buffalo, where heparin and recombinant hyaluronan binding protein 1 (rec-HABP1) were tested for their ability to inhibit sperm binding to the ZP. The inhibitory effect of mannosylated albumin (DMA) could be reversed by rec-HABP1, suggesting competition between rec-HABP1 and DMA demonstrated functional mannose-mediated sperm–ZP recognition [53]. In mice, co-incubation of sperm and oocytes with mannose analogs showed dose-dependent inhibition of sperm binding, supporting the involvement of sperm surface mannosidase in ZP recognition [54].

Dose-dependent inhibition was typically observed, as evidenced by the preincubation of mature porcine oocytes with varying concentrations of a 55 kDa sperm protein. After washing, capacitated sperm were added, and the sperm binding to the ZP was assessed by counting only those attached at the oocyte’s medial plane. Controls included no protein, non-ZP-binding proteins, or elution buffer. Increasing concentrations of the 55 kDa protein resulted in a significant, dose-dependent inhibition of sperm binding to oocytes. At 50 or 100 µg, sperm–oocyte binding was completely blocked, while controls showed no inhibition [27].

In mice, competitive assays were used to study SED1, CRISP1, and ZP3R (also known as sp56). Recombinant SED1 proteins expressed in bacterial and insect cell systems both inhibited sperm–ZP binding in a concentration-dependent manner [32]. Correspondingly, preincubation of mouse oocytes with recombinant rat CRISP1 resulted in a significant inhibition of sperm binding compared to control media or maltose-binding protein [31]. Recombinant ZP3R/sp56, when preincubated with oocytes, also reduced the number of bound sperm [55].

The competition principle was applied in the study of SGG, where artificial liposomes containing SGG were created and added to oocytes in excess. These free SGG-containing liposomes competed with sperm-bound SGG for the same binding sites on the ZP, thereby inhibiting sperm binding [24].

Lectin-based inhibition approaches were also employed. Velásquez et al. [56] demonstrated the role of sialic acids using neuraminidase-treated sperm and sialylated glycoconjugates. Both α2,3-sialic acid-specific lectins (MAA, LFA) and sialylated competitors reduced sperm–ZP binding [56]. This was confirmed by Kashyap et al. [57], who showed that MAA and ABL lectins reduced sperm–ZP binding in a dose-dependent manner. Additionally, Fernandez-Fuertes et al. [58] showed that the removal of sialic acid from bull sperm enhanced their ZP-binding ability, resulting in an increased rate of polyspermic fertilization. Functional blocking of α-L-fucosidase was also achieved using lectin-based competition assays [47]. All studies mentioned in this section are summarized and described in more detail in Table 2.

Competitive binding assays often employ recombinant proteins corresponding to putative sperm receptors. For instance, Yang et al. [22] utilized a recombinant protein to investigate the role of oviductin in hamster fertilization. They demonstrated that a recombinant form of oviductin binds to both sperm and the ZP, and promotes capacitation and the AR. Pre-incubation of either sperm or oocytes with the recombinant protein led to an increase in the number of sperm bound to the oocyte surface, highlighting the importance of oviductin as a key factor in mammalian reproduction [22]. Interestingly, Busso et al. [31] showed that native CRISP1 binds specifically to the ZP, whereas recombinant CRISP1 binds only to the oolemma, suggesting that proper protein conformation is essential for ZP interaction. Deglycosylation of native CRISP1 did not affect ZP binding, but heat denaturation abolished it, highlighting the importance of native folding over glycosylation [31]. This emphasizes that recombinant expression systems have inherent limitations. Even when proteins are produced in mammalian cells, differences in folding or post-translational modifications can alter protein properties compared to their native counterparts [31,59]. Despite these differences, recombinant proteins generally retain essential features such as molecular weight and immunological reactivity, making them valuable tools for various biochemical studies or competitive assays.

Overall, the studies mentioned here illustrate the versatility of competitive binding assays, allowing identification of functionally relevant proteins, analysis of structural specificity (e.g., glycosylation dependence), and discrimination between primary and secondary ZP-binding mechanisms. Sperm–egg competitive binding assays have been widely used to evaluate the functional involvement of sperm proteins and glycoconjugates in ZP binding. Unlike antibody-blocking assays, where sperm are pretreated with antibodies, competitive binding assays involve exogenous proteins or inhibitors added either before or during sperm–egg co-incubation to determine if they occupy or compete for binding sites on the sperm or ZP. These assays help identify functional ligands and receptors, as well as assess structural features (e.g., glycosylation) that are critical for interaction. Advantages of this method include its simplicity, adaptability, and ability to functionally investigate whether a specific antigen competes for the same binding site or binds independently at distinct sites, without directly altering the sperm. However, limitations include non-specific effects at high competitor concentrations, potential alterations to the ZP structure, and a lack of direct information about which molecular interactions are being disrupted. To ensure accurate interpretation, it is crucial to include appropriate controls, such as inactive analogs, identical molecular weight peptides, and untreated groups, as well as to verify that the competitive agent does not impair sperm motility or viability. Results should be interpreted in combination with receptor-blocking or localization studies for a comprehensive understanding. Another constraint of this approach is that synthetic/recombinant antigens may not accurately mimic the natural binding conformation of native proteins, and the absence of native post-translational modifications or membrane environments can influence binding activity. Therefore, combining recombinant protein studies with complementary antibody-blocking or genetic approaches enhances functional validation of sperm–ZP receptor candidates.

### 2.3. Hemizona Binding Assay

Due to ethical limitations, it is not always feasible to incubate human sperm samples with antibodies to study their effects on human reproductive outcomes. As a result, to evaluate the ability of human sperm to firmly bind to the human ZP and thereby predict fertilization potential, the hemizona binding assay (HZA) can be employed to explore the role of different protein receptors in human fertilization, paired with either antibodies or competitors [60]. This method was developed by [61] and further modified for different aims [62,63,64].

**Figure 3:**
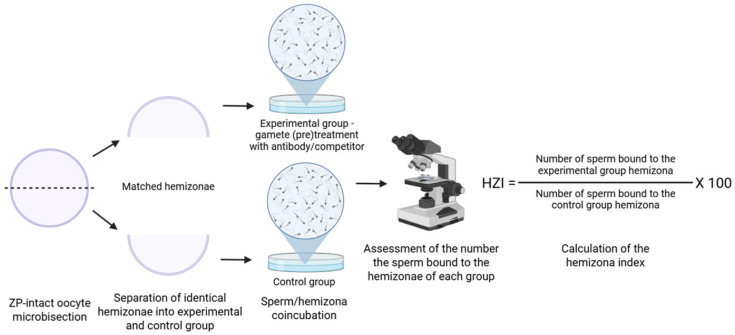
Schematic representation of the hemizona binding assay. Zona pellucida is bisected into two identical halves (dashed line) and paired halves of a single human ZP (hemizonae) are incubated separately with control sperm and sperm treated with antibodies or competitors targeting the candidate receptor, or the treatment takes place during the sperm/hemizona co-incubation. The number of bound sperm is compared between hemizonae, with reduced binding in the experimental group indicating involvement of the targeted molecule in sperm–zona interaction. Created in BioRender. Aneta Pilsova (2025) https://BioRender.com/3veigov (accessed on 30 June 2025).

For this assay, each oocyte is carefully halved using a micromanipulator. The creation of two matched zona hemispheres offers several advantages. The two halves (hemizonae) provide functionally equivalent zona surfaces, allowing for a controlled comparison of sperm binding, the limited availability of human oocytes is amplified, as an internally controlled test can be effectively performed on a single oocyte. Ethical concerns regarding the potential inadvertent fertilization of a viable oocyte are mitigated by initially dividing the egg into halves [61]. The advantage of the HZA is that various sources of human oocytes can also be used, including oocytes obtained from surgically removed ovaries, postmortem ovarian tissue, or excess oocytes from IVF programs [64]. Each hemizona is then separately incubated with test and control groups of spermatozoa (Figure 3). Results are described by the hemizona binding index (HZI), which is the ratio of the number of bound spermatozoa on the test hemizona to that on the control hemizona, multiplied by 100. The limitations of this method include the already mentioned constraints in the number of human oocytes and ethical and legal restrictions [61,64]. HZA results can vary depending on the source of the oocytes, oocyte preservation methods, sperm concentration and preparation technique, media, and diameter of the aspiration pipette [63,65,66,67]. The HZA is often combined with binding inhibition approaches, either using antibodies or through protein or peptide-mediated inhibition [60,68,69].

An antibody block approach, combined with HZA, was employed to validate the ZP-binding protein UBAP2L identified by yeast two-hybrid screening, using anti-UBAP2L antibodies, which resulted in significantly reduced sperm–ZP binding, indicating a functional role [70]. Antibody against galectin-3 also reduced the number of human capacitated sperm bound to hemizonae [71]. In the same way, Wang et al. [72] used HZA to study C1orf56, a Sialyl-Lewis(x)-interacting protein. The Anti-C1orf56 antibody significantly reduced hemizona binding, SLeX binding, and the ZP-induced AR. Recently, Chen et al. [60] showed that anti-SPACA4 antibody reduced sperm binding to hemizona by ~60%. Antibody block with HZA was also used in the analysis of SP-10, where antibody treatment showed no effect [73].

Other HZA studies applied the competitive binding approach, for instance, in the study of zona receptor kinase (ZRK), where a synthetic peptide was used as a competitor and significantly inhibited the sperm/ZP binding [11]. Naz et al. [74] tested the synthetic YLP12 peptide using two HZA strategies, treating either sperm or hemizona. Both treatments significantly reduced sperm binding, confirming functional involvement of YLP12.

Multiple studies have combined these two approaches, using both antibodies and competitor molecules. Kadam et al. [68] demonstrated that antibody blocking fertilization antigen-1 (FA-1) on sperm or pre-treating the ZP with FA-1 significantly inhibited binding, underscoring its role in sperm–ZP interaction. HZA also facilitated the study of sperm fucosyltransferase (FUT). The Anti-FUT antibody reduced sperm binding in a dose-dependent manner, and this effect was reversible with a blocking peptide. Furthermore, FUT acceptors (2-fucosyllactose, lacto-N-fucopentaose I, and asialofetuin) inhibited binding, whereas unrelated sugars (phenyl-β-D-galactoside and melibiose) and RNase had no effect. These treatments did not impair sperm viability, motility, or acrosomal integrity. Anti-FUT5 antibody and its acceptors also reduced sperm binding dose-dependently, but did not completely block binding, likely due to the presence of multiple ZP receptors [69]. Similarly, Maldera et al. [75] investigated human CRISP1 (hCRISP1) using HZA. Anti-hCRISP1 antibody and recombinant hCRISP1 (rec-hCRISP1) both significantly reduced sperm binding without affecting sperm motility, viability, or capacitation. Control protein (MBP) had no effect. Zona-intact oocyte assays confirmed binding specificity: rec-hCRISP1 bound the human ZP, while rat rec-CRISP1 and medium control did not.

As demonstrated here and summarized in Table 3, the HZA has been widely used to assess the functional involvement of various human sperm surface proteins in ZP binding and remains a useful method for studying human sperm–ZP interaction. It provides functionally equivalent zona surfaces, allowing direct comparison of test and control sperm binding under identical conditions. This not only grants proper integral controls but also maximizes the use of limited human oocytes, enabling effective analysis from a single egg. Additionally, ethical concerns related to inadvertent fertilization are mitigated by dividing the oocyte prior to fertilization. HZA is valuable for validating sperm receptors through antibody blocking or ligand competition, as well as assessing sperm function in fertility research. However, the use of this method can be limited by the technical demands of oocyte bisection.

## 3. Visualization Methods

An integral part of studying sperm proteins that bind to the ZP is the diverse range of imaging techniques. The most commonly used method is fluorescence microscopy, which enables the investigation of the presence of specific proteins in cells and their mutual interactions. Light microscopy is frequently employed to observe dynamic processes, ranging from sperm motility and binding to the oocyte to the actual process of fertilization. In contrast, fluorescence microscopy enables the visualization of specific molecular events by using fluorescent markers. In studies of sperm–ZP interactions, fluorescence microscopy can be utilized to assess sperm quality and acrosomal status, as well as to label receptors or cell nuclei. In sperm–oocyte binding studies, light microscopy is routinely used to quantify the number of sperm bound to the oocyte, whereas fluorescence microscopy is commonly applied to visualize the nuclei of bound sperm and oocytes. Immunocytochemistry is also consistently used to localize candidate proteins on sperm surfaces and determine their availability for ZP interaction [76], as it offers high sensitivity. The immunofluorescent detection of sperm proteins on the surface of spermatozoa after sperm capacitation is usually the first indicator that the protein might be involved in the primary sperm ZP interaction [77].

Fluorescence microscopy is used not only for evaluating protein localization, but also for assessing capacitation and the AR. Calcium ionophore is often used to induce acrosomal exocytosis, especially for assessing candidate secondary ZP-binding receptors. Dual labeling with lectins and antibodies can help assess how protein localization changes in relation to acrosomal status [78]. Peanut agglutinin (PNA) is routinely used to assess the sperm acrosomal status and the AR during gamete interaction, since PNA binds solely to the outer acrosomal membrane [79]. In addition, PSA-FITC lectin, which labels the acrosomal content, is often employed [80,81]. In every way, immunofluorescence and flow cytometry are valuable tools for predicting candidate sperm receptors involved in the initial sperm–ZP contact [6].

Among advanced microscopy-based methods, the proximity ligation assay (PLA) stands out for its ability to detect and visualize protein–protein interactions at a nanoscale resolution in situ. PLA uses paired antibodies conjugated to DNA strands, which ligate and amplify signals only when proteins are within close proximity, generating discrete fluorescent dots. This method has successfully identified interactions between the CCT complex and ZPBP2 in mouse sperm [78]. PLA offers distinct advantages by enabling the direct validation of sperm–ZP receptor interactions in intact cells, thereby providing a spatial and subcellular context. This approach complements traditional biochemical assays by correlating protein interactions with functional sperm competence, such as the ability to bind to ZP. Its integration with proteomic analyses strengthens our understanding of the molecular dynamics governing fertilization.

## 4. In Vivo Methods

### 4.1. Genetic Knockout Models

**Figure 4:**
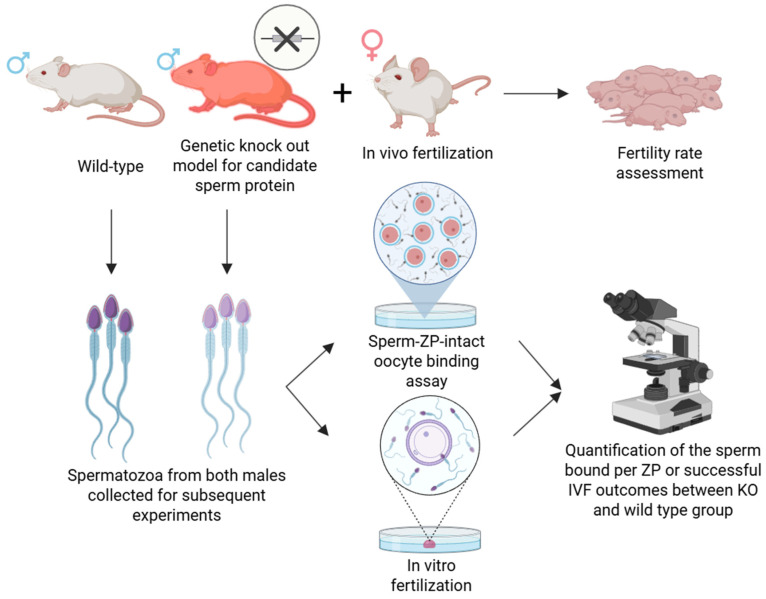
Schematic representation of gene knockout (KO) strategy to investigate candidate sperm receptors involved in zona pellucida binding. Wild-type (white mouse) and gene KO (red mouse; specific gene for a candidate protein is inactivated) males are compared based on the number of pups born following mating, or sperm from wild-type and gene KO males are compared in their ability to bind to the zona pellucida or fertilize zona-intact oocytes. A significant reduction in binding or fertilization efficiency in KO males supports the functional role of the deleted gene product in sperm–zona interaction. Created in BioRender. Aneta Pilsova (2025) https://BioRender.com/ldcgm67 (accessed on 30 June 2025).

In addition to in vitro studies, in vivo approaches are frequently employed to investigate the sperm proteins involved in sperm–oocyte binding, particularly through gene knockout (KO) techniques targeting putative binding proteins. Knockout experiments enable the targeted deletion of genes encoding specific proteins, thereby allowing for the investigation of their in vivo function with higher biological relevance compared to, for instance, recombinant protein studies. Partial or complete gene inactivation allows researchers to assess the role of specific proteins in fertilization. Generally, sperm from KO males is assessed either via in vivo fertilization, IVF assays, or directly in sperm–ZP binding assays (Figure 4). If KO males remain fertile or their sperm exhibit normal binding and fertilization capacity in these assays, it suggests that the targeted protein is either non-essential for ZP binding or its function is compensated for by other proteins. Conversely, infertility or impaired sperm–ZP interaction indicates that the gene plays a critical role in sperm–oocyte binding or fusion. However, these experiments are complex and time-consuming. Moreover, functional redundancy may occur, whereby other proteins compensate for the deleted gene, complicating the interpretation of the gene’s role. These studies are also significantly limited in terms of the animal species used, with the majority being conducted in mouse models [82,83,84].

One of the methods used to generate (KO) models is the CRISPR/Cas9 technology, which employs the Cas9 enzyme guided by a specific sgRNA to locate and cleave a target DNA sequence. Although some of the following proteins are primarily described in the context of sperm–oolemma interaction, the effect of their KO has also been observed to impact sperm binding to the ZP. This genome-editing approach enables the rapid and cost-effective production of KO models and has been applied in the study of SPACA4 [17]. Initial in vitro studies using anti-SPACA4 (SAMP14) antibodies suggested that this protein is involved in both sperm–oocyte binding and fusion [85]. However, SPACA4 KO mice revealed that the protein primarily facilitates sperm penetration through the ZP, rather than membrane binding per se [17], highlighting a functional divergence revealed only through in vivo genetic studies. This mirrors findings for REEP6 KO sperm, which could bind to the ZP but failed to penetrate it [86], again suggesting separable molecular mechanisms for binding and penetration.

An alternative to CRISPR/Cas9 involves introducing a neomycin-tagged DNA construct into embryonic stem cells, where homologous recombination disrupts the target gene. Selected cells are used to create chimeric mice, which are bred to establish knockout lines. This classical gene-targeting strategy has been extensively employed to study the roles of ADAM proteins in fertilization. This classical gene-targeting strategy has been extensively employed to study the roles of ADAM proteins in fertilization. For example, this approach was used to investigate cyritestin (ADAM3), revealing that sperm from KO mice exhibited a marked reduction in binding to the ZP and abnormal expression of multiple sperm proteins, leading to male infertility [18]. It was further demonstrated that sperm binding to the ZP was significantly reduced by the loss of fertilin α (ADAM1a), likely due to the absence of ADAM3 on the sperm surface, which was strongly reduced in ADAM1a-deficient mouse sperm [87]. This research built upon earlier findings concerning fertilin β (ADAM2). In that study, spermatozoa from ADAM2-null mice were found to be incapable of fusing with the oocyte membrane, as expected; however, they also failed to bind to the ZP in in vitro binding assays [84], indicating a broader role in fertilization than initially assumed. Interestingly, further studies have demonstrated that despite the loss of ADAM3, spermatozoa retain fertilizing ability under certain conditions. ADAM3-deficient sperm could successfully fertilize freshly ovulated oocytes when directly deposited into the oviduct, and were also able to fertilize ZP-intact eggs in vitro when the oocytes were surrounded by cumulus cells. Similarly, Adam1a^−^/^−^ sperm, which cannot bind to the ZP, were still capable of fertilizing cumulus-enclosed oocytes in vitro [88].

For instance, SED1, a secreted epididymal protein, is indispensable for initial sperm–ZP adhesion, as SED1-null sperm exhibit severely reduced ZP binding [32]. In contrast, the role of GalTase, a proposed mouse ZP3 receptor and one of the best-studied candidate receptors [7,33], appears more modulatory; although GalTase-null sperm show diminished binding to ZP3 and an impaired ZP3-induced AR, the males remain fertile [83], indicating that GalTase is not an essential mediator.

A further example of the complementary use of in vivo and in vitro approaches is the study of zonadhesin. Zonadhesin KO mice were used to investigate sperm–ZP interaction via sperm–ZP binding assays combined with antibody pretreatment. Capacitated sperm from Zan^−^/^−^ mice displayed increased binding to heterologous ZP, whereas wild-type sperm maintained strict species specificity [89]. In co-insemination experiments, Zan^−^/^−^ sperm consistently outcompeted wild-type sperm for binding to non-mouse ZP. This study elegantly demonstrated that zonadhesin mediates species-specific sperm–ZP adhesion, and its absence leads to broader, non-specific binding, while still allowing fertilization.

Species-specific differences in fertilization mechanisms have also been demonstrated through the in vivo studies. One of the examples is acrosin. While mouse and rat acrosin-null sperm retained ZP binding and variable penetration ability [82,90,91], hamster acrosin-null sperm could bind but failed to penetrate the ZP [92], emphasizing species-specific roles of this enzyme. A conceptual parallel is seen in studies of ZP3R (sp56). Originally thought to be essential for ZP binding [93], KO studies showed no significant reduction in ZP binding [94], highlighting both the limitations of antibody-based in vitro assays and the necessity of genetic validation.

Other proteins, such as CALR3 and TESP5/PRSS21, further enrich this complex picture. CALR3-null sperm exhibit reduced ZP binding in both mice and men [95,96], providing a rare translational bridge between animal models and human infertility. Moreover, TESP5-null sperm exhibit reduced ZP binding [97], reinforcing the notion that epididymal maturation contributes essential components for sperm–ZP interaction.

Knockout experiments enable the targeted deletion of genes encoding specific proteins, allowing for the investigation of their in vivo function with higher biological relevance than studies using recombinant proteins. However, these experiments are complex and time-consuming, and functional redundancy often occurs, whereby other proteins compensate for the deleted gene. Sperm from KO males is evaluated either through in vivo fertilization, IVF assays, or direct sperm–ZP binding assays. Suppose KO males remain fertile or their sperm shows normal binding or fertilization capacity, it suggests that the targeted protein is either non-essential for ZP binding or that other proteins compensate for its function. Conversely, infertility or impaired sperm–ZP interaction indicates that the gene plays a critical role in sperm–oocyte binding or fusion. However, if the studied protein functions as part of a larger multiprotein complex, then its loss could influence the release or activity of other acrosomal proteins during fertilization. Additionally, it is now known that mouse sperm may undergo the AR during passage through the cumulus cell layer before binding to the ZP [98]. Such considerations must be included when evaluating the various study approaches, because most binding assay studies have used cumulus-free oocytes. In contrast, most KO IVF experiments have used cumulus-intact oocytes [94].

Knockout experiments are typically conducted using mouse models, which is a significant limitation [82,83,84]. Although recent advances have enabled the generation of transgenic pigs [99,100], these models have not yet been applied to studies of sperm–ZP binding, primarily due to the time and financial resources required.

In addition to targeted gene KO models, naturally occurring mutations provide valuable opportunities to investigate sperm proteins critical for ZP binding. For instance, a nonsense mutation in TMEM95 identified in Fleckvieh bulls offers compelling evidence. Despite normal sperm motility and morphology, bulls homozygous for this mutation showed drastically reduced fertility, partially due to impaired sperm binding to the ZP [101]. In this study, in vitro-matured oocytes (with cumulus cells removed) were inseminated, and after incubation and vortexing to remove loosely bound sperm, the number of sperm tightly bound to the ZP was counted.

Several other KO studies further underscore the utility of genetic models. These include studies using ACE-null mice [102] and PH-20 hyaluronidase-null mice [103], both of which provide additional insights into sperm function and fertilization.

Gene knockout and in vivo models are essential for validating the functional roles of candidate sperm receptors involved in ZP binding. These models allow assessment of fertility, sperm–ZP binding, and IVF outcomes in a physiologically relevant context, helping to identify critical versus redundant proteins and clarify misleading in vitro findings. While knockout of individual ZP-binding proteins often leads to only partial fertility defects, or none at all, some models reveal infertility due to impaired ZP binding, membrane fusion, or sperm transport. These results suggest that fertilization relies on a network of proteins acting redundantly, synergistically, or in sequence to ensure robust sperm–egg interaction [6].

### 4.2. Other Genetic Methods

Genetic manipulation targeting genes that affect male or female gametes provides valuable insights into fertilization and the initial interaction between gametes. Beyond gene deletions in knockout (KO) models discussed above, genetic approaches also include introducing new genes or mutating existing ones. While KO models are commonly used to study sperm–ZP interactions, transgenic models are also employed for this purpose. Genetic engineering enables the precise insertion of DNA segments into specific loci in the genomes of model organisms, typically mice. For example, transgenic mouse models expressing human ZP proteins have been generated in this manner. These experiments demonstrated that ZP2 plays a key role in mediating sperm–ZP binding [104,105].

In addition to introducing structural proteins, it is also possible to incorporate DNA encoding fluorescent markers into model organisms. An example includes transgenic mice expressing green EGFP fluorescence in the acrosome and red Ds-Red2 fluorescence in the sperm midpiece (mitochondria). This model has been used to study the dynamics of the AR in mice, revealing that sperm undergo acrosomal exocytosis prior to making contact with the ZP [98].

Beyond KO models, sperm–ZP interaction studies can also benefit from other genetic approaches. For instance, the yeast two-hybrid (Y2H) system, combined with validation techniques, has been employed to identify human sperm proteins that interact with the human glycoprotein ZP3. This genetic method enables the identification of proteins that interact with a target protein expressed in yeast as a hybrid with a DNA-binding domain. In one study, human ZP3 cDNA was cloned into the pAS2-1 yeast vector and used as bait in a Y2H screen to identify interacting proteins from a human testis cDNA library. Six specific clones were isolated, and their interactions with ZP3 were further validated using a mammalian two-hybrid system. Sequence analysis revealed that these clones shared homology with various proteins in the GenBank database. The most prominent interactor, showing 97% homology with ubiquitin-associated protein 2-like (UBAP2L), was subsequently evaluated using a HZA [106].

## 5. Discussion

Comparing findings from different studies focusing on candidate sperm receptors for ZP requires cautious evaluation due to the variations in the experimental design and technical parameters. Within individual studies, experimental conditions (i.e., sperm capacitation status, antibody dose/purity, oocyte handling) are typically controlled across experimental groups, offering a valid comparison between them. However, comparisons between studies investigating the same sperm–ZP binding protein but under different assay setups or biological conditions are often problematic and may lead to conflicting interpretations.

The source and quality of antibodies used in sperm–ZP binding assays can strongly influence the reliability and interpretation of results. In-house produced antibodies offer the advantage of being raised against specific epitopes but require thorough validation and consistent purification to avoid non-specific effects that can lead to false positives due to steric hindrance or non-specific IgG, or false negatives due to over-dilution or low-affinity. Especially in older studies, whole antisera were often used, which contain not only the specific antibodies but also a range of non-specific serum proteins that may affect sperm viability, motility, or ZP structure, increasing the risk of non-specific blocking [42,44]. Hybridoma supernatants, which were used, for example, in Margaryan et al. [21], often contain undefined immunoglobulin concentrations and cell culture proteins that may affect sperm viability or bind non-specifically to the ZP. The degree of antibody purification is therefore significant. Crude antiserum or unpurified hybridoma supernatants can contain contaminants and non-specific immunoglobulins that interfere with sperm motility or viability, or cause steric blocking of sperm–ZP interaction. At the same time, affinity-purified antibodies provide greater specificity and concentration accuracy. Fab fragments are used in some studies, i.e., [10,26,74] instead of whole IgG, since they lack the Fc region minimizing non-specific effects such as agglutination. Appropriate isotype, preimmune serum controls, or hybridoma medium controls, as well as testing for sperm viability and motility effects, are essential to distinguish true functional inhibition from artefactual blocking [32,42,44,74]. Commercial antibodies may provide more standardized quality and validation, yet their epitope specificity may be limited, especially for blocking sperm proteins. Moreover, commercial antibodies are often generated against mouse or human proteins, and finding an appropriate antibody cross-reacting with proteins in other experimental models, such as pigs, may be difficult or even impossible.

The sperm capacitation status is another critical factor that may affect the outcomes of the functional sperm–oocyte binding assays, as well as the genetic knockout binding assays. Incomplete capacitation may prevent receptor proteins from being exposed or in the proper conformation, leading to false-negative results. Similarly, premature AR may force spermatozoa to use secondary binding pathways and confound interpretation. Assessment of the sperm capacitation status and control of the capacitation conditions are, therefore, crucial for obtaining reliable results [43].

Likewise, the maturation stage of the oocytes is another key variable, as fully mature metaphase II oocytes possess a structurally and biochemically competent ZP that selectively ensures sperm binding. Although sometimes used, especially in human studies [60], immature oocytes may display an altered ZP composition, leading to non-specific sperm attachment or reduced binding efficiency [107]. This can mask the specific effects of antibodies, competitors, or gene knockouts. Similarly, prolonged storage, excessive pipetting, or suboptimal culture conditions can alter the structural and functional integrity of the ZP. Ideally, sperm–ZP binding assays should be conducted with freshly collected, in vivo-matured MII oocytes, or with carefully validated in vitro-matured MII oocytes, under standardized conditions. Using commercially available glass pipettes with a defined diameter to standardize the washing process and avoid mechanically disturbing and stripping the sperm bound to the ZP is also advisable.

Moreover, although ZP-intact oocytes without cumulus cells are generally used, and the use of cumulus-oocyte complexes in the binding assays is relatively rare [i.e., 45, 58], the presence or removal of cumulus cells may shift the binding pathway used by sperm. Although it has long been believed that sperm undergoes the AR after the sperm primary binding to the ZP, studies in mice have shown that most do so during penetration of the cumulus cells [98]. Therefore, there are two pathways in the sperm binding process to ZP and the AR. The first pathway is that the acrosome-intact sperm binds to the ZP, followed by AR, whereas the second one is that the sperm undergoes AR during the passage of cumulus cells, and the acrosome-reacted sperm bind to the ZP [108]. This is then a notable source of conflicting interpretations. When sperm binding assays are conducted with oocytes lacking cumulus cells, antibodies targeting proteins involved in primary zona pellucida binding may effectively inhibit sperm–ZP interaction. However, such antibodies would not inhibit the fertilization of the eggs with cumulus cells, since the sperm undergoes AR upon contact with the cumulus cells, and the sperm plasma membrane protein would not be present at the contact with the ZP. Since most KO IVF experiments use cumulus-intact oocytes, male mice lacking a specific gene may remain fertile because the AR is induced during sperm penetration of the cumulus cell layer [94].

Selecting the appropriate experimental approach to validate candidate sperm receptors requires careful consideration of the studied protein localization and accessibility or maturation stage-specific expression and dynamics, gamete availability, specific antibody availability, species constraints, assay reproducibility, or technical feasibility. Antibody-blocking assays using cumulus-free oocytes are practical for initial testing whether a candidate sperm protein is directly involved in primary ZP binding, but well-characterized, properly dosed antibodies and appropriate controls are essential. Competitive binding assays can further support receptor-ligand specificity by showing that excess soluble protein can inhibit sperm–ZP binding, when careful titration of the ligand is performed. HZA, while more labor-demanding, are particularly valuable when oocyte availability is limited, especially in human, and offer quantitative paired comparison of treated and control groups. Genetic KO models are the most technically and time demanding, often requiring sophisticated breeding strategies and extensive validation, but provide the most definitive evidence of whether a protein is essential for fertilization in vivo. While most KO models have been established in mice, recent advances in genome editing, particularly CRISPR/Cas9, have started to enable similar approaches in livestock species, especially pigs [109], which are considered both agriculturally and biomedically relevant. However, KO animals may remain fertile if alternative pathways exist due to alternative pathways or backup mechanisms, including protein redundancy or compensatory biological responses. Combining complementary methods while carefully controlling for experimental conditions gives the most reliable information about the role of the studied protein.

## 6. Future Directions

Advances in the study of sperm–ZP interactions have moved the field beyond the classical “lock and key” model, which proposed a single sperm receptor as essential for ZP binding [3]. A growing body of evidence now supports a more complex model involving multiple sperm surface proteins that act cooperatively during the primary recognition and attachment to the oocyte’s extracellular matrix. This conceptual shift has methodological consequences, as identifying and validating such proteins requires an integrative strategy that combines biochemical, molecular, and functional approaches. Functional validation is typically achieved through antibody inhibition or competitive binding assays, which can disrupt protein–ligand interactions and reduce sperm–ZP binding, thereby demonstrating physiological relevance [3,6,15]. Studies have shown that many sperm receptors are not independent actors but components of multimeric recognition complexes assembled on the sperm surface [6]. Disruption of these complexes impairs sperm–ZP interaction, supporting their cooperative function. However, despite the large number of sperm proteins reported to bind the ZP, the precise molecular mechanisms underlying this interaction remain incompletely understood. Tanphaichitr et al. [6] suggested that many proteins were identified under experimental conditions that poorly reflect the in vivo fertilization environment, possibly explaining functional redundancy. Experimental limitations also contribute to this uncertainty. In many species, ethical or practical barriers constrain access to sufficient numbers of mature oocytes and capacitated sperm, especially in humans or endangered species. Fertilization is a dynamic, multistep process that is difficult to replicate in vitro. These challenges underscore the need for more refined experimental models that more closely mimic physiological conditions. While classical in vitro assays laid the foundation for this field by enabling testing of putative sperm–ZP receptors, newer technologies, such as synthetic gamete-mimicking beads, offer promising complementary tools for dissecting sperm–ZP interactions. Future research should focus on integrating classical functional assays with modern genetic approaches to resolve remaining ambiguities about receptor redundancy, multimeric complexes, and species-specific differences. In vitro experiments fail to fully replicate the dynamic and complex environment of fertilization in vivo. Future studies should prioritize developing and adopting experimental systems that more closely mimic physiological conditions, for example, using cumulus-intact oocytes, and fully mature gametes that reflect the physiological maturation stage at the time of sperm–oocyte meeting, or adopting advanced culture systems.

## 7. Conclusions

This overview focused on key experimental strategies used to evaluate candidate sperm receptors, outlining the principles, strengths, and limitations of these methods (summarized in Supplementary Table S1). Because the sperm–ZP interaction relies on the coordinated action of multiple sperm proteins rather than a single receptor, studying this event demands an integrative, physiologically relevant approach. If one message should be taken away from this review, it is that no single assay can conclusively define the functional role of a candidate sperm receptor in ZP binding. Instead, a combination of complementary methods, each chosen appropriately and with awareness of their scope and limitations, provides the most robust evidence. The continued refinement of experimental approaches and innovative tools will be essential for the identification of all the molecules participating in this process.

## Figures and Tables

**Table 1:** Summary of key studies employing antibody-blocking assays to functionally validate putative sperm–ZP receptors.

Antibody Block Binding Assay							
Species	Sperm Source/Preparation	Oocyte Source	Antibody Target (Type of Antibody)	Antibody Co-Incubation Timing	Methodological Advances/Differences	Key Findings	Study
Porcine	Percoll gradient centrifuged capacitated boar sperm	Porcine ZP-intact oocytes	AWN spermadhesin (in-house polyclonal affinity-purified Ab and monoclonal Ab Bo.5)	Sperm/oocyte coincubation (30 min, stopped with NaN3)	Compared mono- vs. polyclonal Ab; highlighted epitope coverage and blocking efficiency differences	All anti-AWN antibodies inhibited sperm–ZP binding; polyclonal Ab blocked ZP binding more effectively (87%) than monoclonal Ab (64%)	Veselský et al. 1999 [20]
Mouse	Caudal epididymal and vas deferens collected Percoll gradient centrifuged mouse sperm	Mouse ZP-intact oocytes	SLIP1 (AS-A) (affinity purified from either whole antiserum or IgG fraction of polyclonal rabbit Ab)	Sperm preincubation (30 min, 1 h) Oocyte preincubation (no effect)	Used dose-dependent response; controls included medium only and the eluate of BSA blots preincubated with anti-SLIP1 of preimmune rabbit serum; only sperm that were in the same focal plane as the diameter of the ZP were counted; two-cell embryos were used as a control for non-specific binding	Anti-SLIP1 antibodies reduced sperm–ZP binding in a dose-dependent manner	Tanphaichitr et al., 1993 [23]
Mouse	Caudal epididymal and vas deferens collected Percoll gradient centrifuged mouse sperm	Mouse ZP-intact oocytes	SGG (in-house generated polyclonal Ab affinity purified from the rabbit antiserum IgG)	Sperm preincubation (30 min)	Used dose-dependent response, highlighted glycolipid role	Anti-SGG Ab reduced sperm–ZP binding in a dose-dependent manner, identifying SGG as a key ZP-binding molecule	White et al., 2000 [24]
Human	Capacitated human sperm (infertility clinic)	Human ZP-intact oocytes failed fertilization at IVF clinic	SLIP1 (rabbit polyclonal Ab in-house generated against rat testis SLIP1 and purified by SGG affinity chromatography)	Sperm preincubation (30 min)	Controlled for sperm function (motility, sperm acrosome status); species translation of method; normal rabbit serum IgG used as control	Anti-SLIP1 IgG reduced sperm–ZP binding; effect not due to motility or acrosome status	Rattanachaiyanont et al., 2001 [25]
Mouse	Caudal epididymal and vas deferens collected Percoll gradient centrifuged mouse sperm	Mouse ZP-intact oocytes	AS-A (IgG, Fab, affinity-purified IgG)	Sperm preincubation (30 min)	Compared IgG vs. Fab to test for steric hindrance (anti-AS-A Fab fragments also showed similar inhibitory effects)	Anti-AS-A IgG and Fab reduced binding up to 70%; similar effects for affinity-purified and Fab	Tantibhedhyang-kul et al., 2002 [10]
Porcine	Percoll gradient centrifuged capacitated boar sperm	Porcine ZP-intact oocytes	AS-A (rabbit polyclonal Ab; IgG, Fab)	Sperm preincubation (45 min)	Cross-species validation; compared IgG and Fab effects	Anti-AS-A Antibodies inhibited sperm–ZP binding in a dose-dependent manner; Fab fragments also inhibitory	Carmona et al., 2002 [26]
Porcine	Capacitated boar sperm	Porcine ZP-intact oocytes	55 kDa sperm protein (in-house generated mouse polyclonal Ab)	Sperm preincubation (1 h before completing capacitation period)	Titration of antibody; established threshold for blocking effect	Anti-55 kDa Ab reduced binding in a dose-dependent manner; complete block at 1:100 dilution	Zayas-Pérez et al., 2005 [27]
Porcine	Percoll gradient centrifuged capacitated boar sperm	Porcine ZP-intact oocytes	pB1/DQH (in-house generated mouse monoclonal Abs, DQH/G12 and DQH/H6)	Both sperm preincubation (30 min) and sperm/oocyte coincubation (30 min, stopped by NaN3)	Compared sperm vs. sperm/oocyte treatment; spermatozoa preincubated in ascites of myeloma cells Sp2/0 and without any pretreatment were used for controls	DQH antibody blocked sperm–ZP binding in both experiments (sperm and sperm/oocytes coincubation)	Maňásková et al., 2007 [29]
Porcine	Capacitated boar spermatozoa	Porcine ZP-intact oocytes	Acrosin (monoclonal Ab ACR.2); Human intra-acrosomal protein (in-house generated mouse monoclonal Ab Hs-8)	Both sperm preincubation (30 min) and sperm/oocyte coincubation (30 min)	Timing-specific inhibition analysis; showed inhibition only during co-incubation, not pre-treatment; probed AR-released proteins; spermatozoa after induction of AR were used as a negative control for the binding test	Both antibodies reduce the secondary sperm–ZP-binding. Hs-8 blocked sperm–ZP binding only when present during incubation, not during sperm pre-treatment	Peknicova et al., 2001 [30]
Porcine	Capacitated boar sperm	Porcine ZP-intact oocytes	GAPDHS (in-house generated monoclonal Ab Hs-8: undiluted hybridoma supernatant; anti-GAPDHS: commercial monoclonal Ab)	Sperm/oocyte coincubation (30 min—stopped by NaN3)	Used both lab-produced and commercial Ab; monoclonal Ab against progesterone, acrosin (GAPDHS is an intra-acrosomal protein), or medium only used as a control group	Both antibodies reduced sperm–ZP binding	Margaryan et al., 2015 [21]
Mouse	Capacitated mouse sperm	Mouse ZP-intact oocytes	CRISP1 (rabbit polyclonal Ab)	Sperm preincubation (30 min)	Dilution series; used normal rabbit IgG and medium only as controls	Anti-CRISP1 Ab reduced sperm–ZP binding in a dilution-dependent manner	Busso et al., 2007 [31]
Mouse	Capacitated mouse sperm	Mouse ZP-intact oocytes	SED1 (Purified anti-SED1 IgG)	Sperm/oocyte coincubation (30 min)	Used preimmune IgG as a control, and two-cell embryos as negative controls	Anti-SED1 drastically reduced sperm–ZP adhesion	Ensslin & Shur, 2003 [32]
Bovine	Fresh bull sperm	Bovine ZP-intact oocytes	GalTase (rabbit antiserum)	Sperm preincubation (1 h)	Used antiserum against recombinant protein; SEM for quantification, quantification of sperm bound to the upper hemisphere of the oocyte	Anti-GalTase antiserum reduced sperm binding to oocytes (SEM quantification)	Tengowski et al., 2001 [34]
Human, Mouse, Porcine	Swim up selected capacitated human sperm; Percoll gradient centrifuged capacitated boar sperm; caudal epididymal swim up selected capacitated mouse sperm	Human, mouse, and porcine ZP-intact oocytes	Human acrosomal antigen (1A1—monoclonal Ab)	Sperm/oocyte coincubation (up to 12 h for IVF)	Cross-species application; tested IVF impact; medium only or the supernatants of other antibodies (e.g., anti-ZP, anti-hCG) used as controls	1A1 Ab reduced sperm binding to ZP and IVF rates across species.	Dubova-Mihailova et al., 1991 [35]
Bovine	Pooled frozen-thawed Percoll gradient centrifuged bull sperm, capacitated during sperm/egg interaction	Bovine ZP-intact oocytes	SPAM1 (monoclonal antibody (203–7D10): N-terminal domain-specific Ab; or custom-made rabbit polyclonal C-terminal domain-specific Ab)	Sperm/oocyte coincubation (6 h)	Domain-specific blocking; mapped functional region of SPAM1; mouse or rabbit IgG used as controls	Blocking C-terminal domain reduced binding by 85%, N-terminal by 30%	Morin et al., 2010 [36]
Guinea pig	Acrosome-reacted guinea pig sperm	Guinea pig ZP-intact oocytes	PH-20 (in-house generated mouse monoclonal Ab)	Sperm preincubation during the induction of AR	Focused on secondary ZP receptors; used in vitro AR prior to assay; medium only used as a control; sperm counted in a single plane of focus	Anti-PH-20 antibody blocked binding of acrosome-reacted sperm to ZP	Primakoff et al., 1985 [37]
Bovine	Frozen-thawed Percoll gradient centrifuged bull sperm, capacitated and acrosome reacted during coincubation with oocytes	Bovine ZP-intact oocytes	SP-10 (monoclonal Ab cocktail containing monoclonal antibodies MHS-10, 6C12, and 3C12 targeting human SP-10, and rabbit polyclonal antisera raised against a recombinant SP-10 fusion protein)	Sperm/oocyte coincubation (18 h)	Used staged washing (pipetting, vortex) to distinguish superficial vs. stable binding; control antibodies or medium only as controls	SP-10 antibodies interfered with stable secondary sperm–ZP binding; only superficial attachment observed	Coonrod et al., 1996 [38]
Human	Capacitated human sperm	Human ZP-intact oocytes	CCT6A, ZPBP2 (Ab not specified)	Sperm preincubation (30 min)	Assayed protein complexes, not just single proteins; compared native lysate preincubation	Ab block of protein complexes reduced sperm–ZP binding	Redgrove et al., 2011 [39]
Mouse	Caudal epididymal capacitated mouse sperm	Mouse ZP-intact oocytes	sp56 (Ab not specified)	Sperm preincubation (15 min), then sperm/oocyte coincubation (additional 30 min) Alternatively, oocytes preincubated with sperm membrane vesicles (15 min)	Used sperm membrane vesicles; compared vesicle and whole-cell blocking—sperm vesicles were used for antibody block, and their preincubation with anti-sp56 also showed a 6–7-fold reduction in their ability to block sperm–egg binding; medium only and two-cell embryos used as a control	Anti-sp56 reduced sperm–ZP binding by 70–80%; vesicle block confirmed functional role	Cohen & Wassarman, 2001 [40]
Human	Capacitated swim-up selected human sperm (infertility clinic)	Human ZP-intact oocytes	C5F10 (anti-proacrosin/acrosin, monoclonal Ab)	Sperm pretreatment (15 min) and then gamete coincubation (additional 3 h)	Used functional controls to rule out non-specific detrimental effects of the antibody on spermatozoa (fertilization in hamster eggs)	C5F10 inhibited sperm–ZP binding; the effect was not due to sperm dysfunction	Moreno et al., 1998 [41]
Mouse	Caudal epididymal mouse spermatozoa	Mouse ZP-intact oocytes	HABP (polyclonal antiserum)	Sperm preincubation (30 min)	Standard antibody-blocking assay; functional validation-tested for agglutination caused by the antiserum; preimmune rabbit serum used as a control	Ab block reduced sperm–ZP binding, implicating HABP role in the binding	Ranganathan et al., 1994 [42]
Porcine	Percoll gradient centrifuged boar sperm	Porcine ZP-intact oocytes	ACRBP (Ab not specified)	Sperm preincubation and sperm/oocyte coincubation	Recent application; standard functional block; medium only, and pre-immune rabbit IgG used as controls	Ab block reduced sperm–ZP binding, implicating ACRBP role in the binding; anti-ACRBP Ab also impeded capacitation and the AR	Kato et al., 2021 [43]
Human	Percoll gradient centrifuged human sperm	Unfertilized human ZP-intact oocytes	P34H/P26h (antiserum)	Sperm preincubation (1 h)	Cross-species validation; standard Ab-blocking, preimmune serum used as a control; tested for the effect of the antibody on the sperm acrosome status and motility	Ab block reduced sperm–ZP binding. The inhibition was not due to the induction of premature AR nor to an effect on the motility of the spermatozoa	Boue et al., 1994 [44]
Hamster	Caudal epididymal capacitated hamster sperm	Cumulus cell oocyte complexes	P34H/P26h (preimmune serum)	Sperm/oocyte coincubation (35 min)	Sperm selected based on hyperactivation. COCs used, cumuli removed after gamete coincubation	Ab block reduced sperm–ZP binding	Berube & Sullivan, 1994 [45]
Hamster Mouse	Caudal epididymal capacitated hamster and mouse sperm	Hamster and mouse ZP-intact oocytes	P34H/P26h (IgG and Fab fragments of polyclonal Ab)	sperm/oocyte coincubation (35 min)	Compared cross-reactivity of mouse and hamster sperm proteins recognized by the antibody against hamster P34H (P26h)	IgG reduced sperm–ZP binding in both hamster and mouse, although the reduction was less significant in mouse, Fab fragments only in hamster	Bégin et al., 1995 [46]
Mouse	Caudal epididymal capacitated mouse sperm	Mouse ZP-intact oocytes	α-L-fucosidase (Ab not specified)	Sperm preincubation (30 or 60 min)	Functional block; 30 and 60 min of gamete co-incubation—data were plotted as average number of sperm tightly bound to the ZP per oocyte versus length of gamete co-incubation for each group; IgG used as a control	Ab block reduced sperm–ZP binding	Phopin et al., 2013 [47]
Mouse	Caudal epididymal and vas deferens Percoll gradient centrifuged mouse sperm	Mouse ZP-intact oocytes	SLIP1/AS-A (polyclonal Ab-Fab fragments)	Sperm preincubation (30 min)	Functional block; confirmed previous findings. Nonimmune rabbit serum Fab, and two-cell embryos were used as a control	Ab block reduced sperm–ZP binding, implicating SLIP1/AS-A	Moase et al., 1997 [48]

Ab—antibody; ACRBP—Acrosin-Binding Protein; AR—acrosome reaction; CCT6A—Chaperonin Containing TCP1 Subunit 6A; GalTase—β-1,4-galactosyltransferase; GAPDHS—Glyceraldehyde-3-phosphate dehydrogenase, sperm-specific; HABP—Hyaluronic Acid Binding Protein; PH-20—hyaluronidase; SED1—Secreted Epididymal protein 1 (known as lactadherin); SGG—sulfogalactosylglycerolipid; SLIP1 (AS-A)—arylsulfatase; SPAM1—Sperm Adhesion Molecule 1 (known as PH-20, hyaluronidase), ZP—zona pellucida; ZPBP2—zona pellucida binding protein 2.

**Table 2:** Summary of key studies employing competitive binding assays to validate the function of putative sperm–ZP receptors.

Competitive Binding Assay							
Species	Sperm Type and Preparation	ZP Source	Competitor	Competitor Co-Incubation Timing	Methodological Advances	Key Findings	Study
Porcine	Capacitated boar sperm	Porcine ZP-intact oocytes	Isolated purified AQN-1 (15 kDa protein from boar ejaculated spermatozoa)	Oocyte preincubation (1 h)	Introduced concentration-dependence; used native protein receptor; medium only as a control	AQN-1 reduced sperm binding in a dose-dependent manner	Veselský et al., 1992 [51]
Porcine	Capacitated boar sperm	Porcine ZP-intact oocytes	Isolated purified AWN-1,2/AQN-1 (from boar ejaculated spermatozoa)	Oocyte preincubation (1 h)	Established minimum inhibitory concentration protocol; compared multiple spermadhesins	All tested spermadhesins significantly inhibited sperm binding in a dose-dependent manner, suggesting critical roles in ZP recognition (at ≥50 µg/mL)	Sanz et al., 1992a,b [19,52]
Porcine	Percoll gradient centrifuged capacitated boar sperm	Porcine ZP-intact oocytes	pB1/DQH antigen isolated from boar seminal plasma	Sperm/oocyte coincubation	Co-incubation was stopped by the addition of 50 μL of 10% NaN_3_ solution into the droplets containing the gametes	Sperm binding was strongly reduced, the percentage of ZP-bound spermatozoa decreased to 14%	Maňásková et al., 2007 [29]
Mouse	Percoll-gradient centrifuged sperm	Mouse ZP-intact oocytes and isolated ZP	Native SLIP1 (AS-A, isolated from rat testis, and mouse epididymal and vas deferens sperm)	Both oocyte pre-incubation (30 min) and sperm/oocyte coincubation (30 min) Sperm/isolated ZP coincubation (10 min)	Biotinylated SLIP1 was used for verification of the binding to the ZP. SLIP1 denaturation experiments to confirm loss of function	Inhibition required native SLIP1 conformation; SLIP1 binds ZP; the antibody blocked AR	Tanphaichitr et al., 1993 [23]
Bovine	Pooled frozen-thawed Percoll gradient centrifuged bull sperm, capacitated during sperm/egg interaction	Bovine ZP-intact oocytes	Purified SPAM1 (from bull sperm, deglycosylated vs. native)	Oocyte preincubation (2 h)	Dose-dependent inhibition; glycosylation status analysis, used native, total, N-deglycosylated, or O-deglycosylated bull sperm SPAM1, or deglycosylation buffer and enzymes as controls	Dose-dependent reduction in sperm binding. Deglycosylation of SPAM1 did not alter its inhibitory effect on sperm–ZP binding, demonstrating that SPAM1-mediated binding is independent of its glycosylation status	Morin et al., 2010 [36]
Water buffalo	Frozen-thawed Percoll gradient centrifuged capacitated water buffalo sperm	Water buffalo ZP-intact oocytes	Recombinant HABP1 (*E. coli* expressed) + DMA (synthetic)	Sperm/oocyte coincubation (2 h)	Competitor reversal protocol used—DMA considered a substitute for ZP	Sperm surface HABP1 may serve as mannose-binding sites for zona recognition	Ghosh & Datta, 2003 [53]
Mouse	Caudal epididymal mouse sperm	Mouse ZP-intact oocytes	Mannose analogs	Sperm/oocyte coincubation (1 h)	Inhibition study using various sugars; tested for deleterious effects of the mannose analogs on sperm motility and acrosome status, none were observed	Dose-dependent inhibition of sperm–egg binding and mannosidase activity implicated the involvement of a sperm surface mannosidase in ZP recognition	Cornwall et al., 1991 [54]
Porcine	Capacitated boar sperm	Porcine ZP-intact oocytes	55 kDa protein (mouse testis, unlabeled, ion-exchange purified)	Oocyte preincubation (1 h)	Medial plane quantification method; controls included no protein, non-ZP-binding proteins, or elution buffer	55 kDa protein caused dose-dependent inhibition; Complete inhibition at ≥50 µg	Zayas-Pérez et al., 2005 [27]
Mouse	Capacitated mouse sperm	Mouse ZP-intact oocytes	Recombinant SED1 (His-tagged expressed in *E. coli*/GST-fusion expressed in insect cells, purified)	Addition during sperm–ZP interaction (30 min)	Dual expression system validation	Both recombinant protein forms showed dose-dependent inhibition	Ensslin & Shur, 2003 [32]
Mouse	Capacitated mouse sperm	Mouse ZP-intact oocytes	Purified native rat CRISP1; bacterially expressed recombinant CRISP1	Oocyte preincubation (30 min)	Cross-species protein testing; maltose-binding protein or medium alone used as a control	Conserved CRISP1–ZP interaction across rodents. Native rat CRISP1, but not recombinant CRISP1, interfered with the sperm–ZP interaction	Busso et al., 2007 [31]
Mouse	Caudal epididymal mouse sperm	Mouse ZP-intact oocytes	Recombinant ZP3R/sp56	Oocyte preincubation (45 min)	Two-cell embryos used as negative controls	Specific inhibition of sperm binding to ZP-intact oocytes	Buffone et al., 2008 [55]
Mouse	Caudal epididymal and vas deferens Percoll gradient centrifuged pre-capacitated mouse sperm	Isolated ovarian ZP	SGG-liposomes (synthetic, cholesterol:SGG 1:1)	Sperm/oocyte co-incubation (10 min)	Artificial membrane system development	Free SGG-containing liposomes competed with sperm-bound SGG for the same binding sites on the ZP, thereby inhibiting sperm binding	White et al., 2000 [24]
Bovine	Frozen-thawed Percoll gradient centrifuged	Isolated bovine ZP	Sialic acid-specific lectins (LFA, MAA, and SNA)	Oocyte preincubation (1 h)	Lectin specificity profiling; combined binding competition assays and IVF in combination with different lectins, and neuraminidase digestion	MAA lectin recognizing a-2,3-linked sialic acid and neuraminidase with catalytic activity for a-2,3-linked sialic acid, demonstrated a high inhibitory effect on the sperm–ZP binding, indicating the presence of a sperm plasma membrane-specific protein for the sialic acid	Velásquez et al., 2007 [56]
Buffalo	Pooled swim-up selected buffalo sperm	Buffalo ZP-intact oocytes	MAA/ABL lectins (commercial, FITC-conjugated)	Oocyte preincubation (1 h)	Sperm selection via density gradient	Treatment of ZPs with 25 mg/mL lectin concentration significantly reduced the number of sperm that attached to the ZP	Kashyap et al., 2023 [57]
Bull	Frozen-thawed and Percoll gradient centrifuged bull sperm	Bovine ZP-intact oocytes (some with cumulus cells)	Neuraminidase (*C. perfringens*, 0.1 U/mL)	Sperm preincubation (1 h)	Viability-controlled enzyme treatment; two groups of oocytes—one was stripped of cumulus cells, and the second group was placed in fertilization media without removing their cumulus cells; combined with IVF experiments	Treatment with neuraminidase increased the number of sperm bound to the ZP but also the rate of polyspermic fertilization	Fernandez-Fuertes et al., 2018 [58]
Mouse	Caudal epididymal capacitated mouse sperm	Mouse ZP-intact oocytes	Purified human liver a-L-fucosidase	Oocyte preincubation (30 min)	Use of the enzyme as a competitor	Mouse oocytes preincubated with purified human liver a-L-fucosidase presented a reduced number of sperm tightly bound to the ZP	Phopin et al., 2013 [47]

AR—acrosome reaction; DMA—D-mannosylated albumin; HABP—Hyaluronic Acid Binding Protein; SED1—Secreted Epididymal protein 1 (known as lactadherin); SGG—sulfogalactosylglycerolipid; SLIP1 (AS-A)—arylsulfatase; SPAM1—Sperm Adhesion Molecule 1 (known as PH-20, hyaluronidase), ZP—zona pellucida.

**Table 3:** Summary of key studies employing the hemizona binding assay to validate the putative sperm–ZP receptors functionally.

Hemizona Binding Assay							
Species	Hemizona Source and Preparation	Sperm Source and Preparation	Studied Protein	Gamete Pretreatment Details	Methodological Advances/Differences	Key Finding	Study
Human	Unfertilized oocytes from the assisted reproduction program	Motile sperm from fertile donors	UBAP2L (ubiquitin-associated protein-2 like)	Sperm preincubated with Ab against UBAP2L (4 h)	Used BSA and control immunoglobulins as controls	Anti-UBAP2L antibody inhibited sperm–ZP binding	Naz & Dhandapani, 2010 [70]
Human	Unfertilized oocytes from assisted reproduction program	Capacitated human sperm	Galectin-3	Sperm preincubation with anti-galectin 3 (1 h)	Irrelevant mouse IgG or mannotriose (a glycan without binding affinity to galectin-3) was used as a control	Ab block reduced sperm–ZP binding, proposing galectin-3 as a sperm–ZP receptor	Mei et al., 2019 [71]
Human	Unfertilized oocytes	Swim up selected capacitated spermatozoa	C1orf56 (chromosome 1 open reading frame 56—SLeX-binding protein)	Sperm preincubated with anti-C1orf56 antibody (1 h)	Non-specific rabbit IgG and medium were used only as controls; linked receptor inhibition to AR; checked for the effects of the antibody on the viability, motility, and acrosomal status	Anti-C1orf56 reduced sperm binding to ZP and the AR	Wang et al., 2021 [72]
Human	Immature oocytes	Swim up selected capacitated spermatozoa	SPACA4 (Sperm Acrosome Associated 4)	Sperm preincubated with anti-SPACA4 antibody	Demonstrated functional inhibition by anti-SPACA4 antibody; checked for the effects of the Ab on the viability, motility, and acrosomal status	After the Ab pretreatment, sperm binding to hemizona was reduced by ~60%	Chen et al., 2023 [60]
Human	Unfertilized oocytes	Swim up selected motile sperm from fertile men	SP-10 (acrosomal protein)	Anti-SP-10 antibody applied	Used an antibody against intra-acrosomal protein	Anti-SP-10 had no effect on sperm–ZP binding	Hamatani et al., 2000 [73]
Human	Unclassified oocytes	Capacitated human sperm	ZRK (zona receptor kinase, 95 kDa protein)	Hemizonae preincubated with synthetic peptides corresponding to various regions of ZRK subsequently incubated with sperm (30 min)	Used competition peptides to evaluate functional receptor role; medium alone was used as a control	Synthetic peptides inhibited sperm binding	Burks et al., 1995 [11]
Human	Oocytes from women without any ovarian abnormality	Swim up selected motile sperm from fertile men	YLP12 (human sperm peptide sequence)	Either sperm or hemizona preincubated with YLP12 peptide or anti-YLP12 Fab’s (both 1 h)	Evaluated both sperm and ZP side interventions with synthetic peptides and Fab’s; checked if peptides or Fab’s caused agglutination of sperm or any apparent deleterious effect on motility; control Fabs from preimmune serum and control animals immunized with the tetanus toxoid (conjugated to peptides) alone used as controls	Both sperm and hemizona treatment with peptide/Fab significantly reduced binding; immunoadsorption of the anti-YLP12 Fabs with purified peptide completely abolished inhibitory activity	Naz et al., 2000 [74]
Human	Oocytes that failed to fertilize during IVF	Motile sperm; preincubated with FA-1 antigen, anti-FA-1 Fab’, control Fab’/BSA	FA-1 (fertilization antigen)	Either sperm or hemizona preincubated with FA-1 antigen/anti-FA-1 Fab’ fragments (1 h)	Tested both sperm and hemizona pretreatment; used BSA and control Fab’ for controls	Anti-FA-1 Fab’ blocked sperm–ZP binding; FA-1 antigen on hemizona blocked binding; controls had no effect	Kadam et al., 1995 [68]
Human	Unfertilized oocytes from the assisted reproduction program	Capacitated human sperm	FUT5 (α1,3/4-fucosyltransferase 5)	Hemizonae together with sperm coincubated with anti-FUT5 antibody or FUT acceptors (3 h)	Used a panel of FUT ligands and antibodies; examined specificity and reversibility of inhibition; antibody preabsorbed with 1:100 blocking peptide was used as control; ability of different concentrations of FUT acceptors to inhibit spermatozoa-zona pellucida binding was also studied; the effect of an anti-FUT antibody and FUT acceptors on sperm motility, viability, and acrosomal status was determined	Anti-FUT5 antibody inhibited the binding in a dose-dependent manner, and the effects were reversible with blocking peptide; some of the FUT acceptors inhibited sperm–ZP binding	Chiu et al., 2007 [69]
Human	Unfertilized or in vitro matured human oocytes	Swim-up selected capacitated sperm	CRISP1 (Cysteine-Rich Secretory Protein 1)	Sperm preincubated with anti-CRISP1 or recombinant human CRISP1 (rec-hCRISP1; 30 min); or hemizonae preincubated with rec-hCRISP1	MBP and normal rabbit IgG were used as controls; confirmed receptor localization and specificity	Anti-hCRISP1 and rec-hCRISP1 inhibited sperm binding; rec-hCRISP1 specifically bound ZP	Maldera et al., 2014 [75]

## Data Availability

Not applicable.

## Supplementary Materials

The following supporting information can be downloaded at: https://www.mdpi.com/article/10.3390/mps8040095/s1, Table S1: Summary of key experimental strategies used to functionally evaluate candidate sperm–ZP receptors, highlighting their advantages, limitations, and the main proteins assessed in each assay.
