# Supplementary material for: Functional Methods for Studying Sperm–Zona Pellucida Interaction in Mammals
Source: Methods Protoc. 2025 Aug 13;8(4):95. doi: 10.3390/mps8040095 (PMC12388836; doi:10.3390/mps8040095)
Supplement: Supplementary file 1 [file mps-08-00095-s001.zip › mps-3758844-supplementary.pdf]

methods and protocols

MDPI

Supplementary Material

Review

Functional Methods for Studying Sperm-Zona Pellucida Interaction in Mammals

Natalie Zelenkova [1], Veronika Kraus [2], Alexandra Maresova [1], Zuzana Pilsova [1], Aneta Pilsova [1], Barbora Klusackova [1], Eva Chmelikova [1], Katerina Komrskova [2,3] and Pavla Postlerova [1,2,*]

[1] Department of Veterinary Sciences, Faculty of Agrobiology, Food and Natural Resources, Czech University of Life Sciences Prague, 165 00 Prague, Czech Republic; zelenkovan@af.czu.cz (N.Z.), xmara142@studenti.czu.cz (A.M.), pilsovaz@af.czu.cz (Z.P.), pilsova@af.czu.cz (A.P.), klusackovab@af.czu.cz (B.K.), chmelikova@af.czu.cz (E.C.)

[2] Laboratory of Reproductive Biology, Institute of Biotechnology of the Czech Academy of Sciences, BIOCEV, 252 50 Vestec, Czech Republic; veronika.kraus@ibt.cas.cz (V.K.), katerina.komrskova@ibt.cas.cz (K.K.)

[3] Department of Zoology, Faculty of Science, Charles University, 128 00 Prague, Czech Republic

* Correspondence: postlerova@af.czu.cz or pavla.postlerova@ibt.cas.cz

Supplementary Table S1. Summary of key experimental strategies used to functionally evaluate candidate sperm–ZP receptors, highlighting their advantages, limitations, and the main proteins assessed in each assay.

Method	Advantages	Disadvantages	Studied proteins
		**Functional Binding Assays**	
In vitro **Binding Assay – Antibody Block**	Specificity<br>Direct gamete interaction<br>Functional relevance<br>Quantifiable<br>Accessible technique<br>Versatility	Steric hindrance<br>Variable antibody quality<br>Potential sperm impairment<br>Interpretation complexity<br>Not suitable for human gametes	**AWN** (Veselský et al. 1999) [20]<br>**SLIP1/AS-A** (Tanphaichitr et al., 1993; Moase et al., 1997; Rattanachaiyanont et al., 2001; Tantibhedhyangkul et al., 2002; Carmona et al., 2002) [10, 23, 25, 26, 48]<br>**SGG** (White et al., 2000) [24]<br>**55 kDa protein** (Zayas-Perez et al., 2005) [27]<br>**pB1/DQH** (Maňásková et al., 2007) [29]<br>**GAPDHS** (Peknicova et al. 2001; Margaryan et al., 2015) [21, 30]<br>**CRISP1** (Busso et al., 2007) [31]<br>**SED1** (p47/lactadherin/MFGE8; Ensslin & Shur, 2003) [32]<br>**GalTase** (Tengowski et al., 2001) [34]<br>**human acrosomal antigen** (Dubova-Mihailova et al., 1991) [35]<br>**PH-20** (SPAM1) (Primakoff et al., 1985; Morin et al., 2010) [36, 37]<br>**SP-10** (Coonrod et al., 1996) [38]<br>**CCT6A, ZPBP2 complexes** (Redgrove et al., 2011) [39]<br>**Sp56** (Cohen & Wassarman, 2001) [40]<br>**Proacrosin/acrosin** (Moreno et al., 1998) [41]<br>**HABP** (Ranganathan et al., 1994) [42]<br>**ACRBR** (Kato et al., 2021) [43]<br>**P34H** (Bégin et al., 1995; Boue et al., 1994) [44, 46]<br>α-**L fucosidase** (Phopin et al., 2013) [47]

***In vitro* Binding Assay – Competitive Binding**	Specificity<br>Direct gamete interaction<br>Functional relevance<br>Quantifiable<br>Mimics receptor involvement<br>Accessible technique<br>Versatility	Steric hindrance<br>Non-specific effects<br>Requires high-affinity competitors	**AQN-1, AWN-1,2** (Sanz et al., 1992a,b; Veselský et al., 1992) [19, 51, 52]<br>**pB1/DQH (**Maňásková et al., 2007) [29]<br>**SLIP1/AS-A** (Tanphaichitr et al., 1993, 1998) [23, 50]<br>**SPAM1** (Morin et al., 2010) [36]<br>**HABP1** (Ghosh & Datta, 2003) [53]<br>**Mannosidase** (Cornwall et al., 1991) [54]<br>**55-kDa protein** (Zayas-Perez et al., 2005) [27]<br>**CRISP1** (Busso et al., 2007) [31]<br>**ZP3R/sp56** (Buffone et al., 2008) [55]<br>**SGG** (White et al., 2000) [24]<br>**Sialic acid** (Vélasquez et al., 2007; Kashyap et al., 2023), (Fernandez-Fuertes et al., 2018) [56–58]<br>**α-L fucosidase** (Phopin et al., 2013) [47]<br>**Oviductin** (Yang et al., 2015) [22]
**Hemizona Binding Assay**	Suitable for studies on human<br>High specificity<br>Functional relevance<br>Integrally controlled<br>Quantifiable	Labor-intensive<br>Time consuming<br>Lack of human ZP	**UBAP2L** (Naz & Dhandapani, 2010) [70]<br>**Galectin 3** (Mei et al., 2019) [71]<br>**Sialyl-Lewis(x) interacting protein** (Wang et al., 2021) [72]<br>**SPACA4** (Chen et al., 2023) [60]<br>**SP-10** (Hamatani et al., 2000) [73]<br>**ZRK** (Burks et al., 1995) [11]<br>**YLP12 peptide** (Naz et al., 2000) [74]<br>**FA-1** (Kadam et al., 1995) [68]<br>**FUT** (Chiu et al., 2007) [69]<br>**CRISP1** (Maldera et al., 2014) [75]
***In vivo* Genetic Methods**			
**Knockout Experiments**	*In vivo*<br>Direct impact<br>Physiological relevance<br>Functional validation<br>Discovery of enexpected roles<br>Multiple KO possible	Expensive<br>Time-consuming<br>Ethical concerns<br>Limited to known genes<br>Possible compensatory mechanisms<br>Low throughput	**SPACA4** (Fujihara et al., 2021) [17]<br>**Reep6** (Devlin et al 2020) [86]<br>**ADAM1,3** (fertilin α, cyritestin; Cho et al., 1998; Nishimura et al., 2001, 2004; Tokuhiro et al., 2012) [18, 84, 87, 88]<br>**SED1** (Ensslin & Shur, 2003) [32]<br>**GalTase** (Lu & Shur, 1997) [83]<br>**Zonadhesin** (Tardif et al., 2010) [89]<br>**Acrosin** (Baba et al., 1994), (Adham et al., 1997; Isotani et al., 2017; Hirose et al., 2020) [90–92]<br>**Sp56/ZP3R** (Muro et al., 2012) [94]<br>**Calreticulin 3** (Ikawa et al., 2011) [96]<br>**Prss21/Tesp5** (Yamashita et al., 2008) [97]<br>**ACE** (Hagaman et al., 1998) [102]<br>**PH-20** (Baba et al., 2002) [103]
